# Seed ejection mechanism in an *Oxalis* species

**DOI:** 10.1038/s41598-020-65885-2

**Published:** 2020-06-01

**Authors:** Shanpeng Li, Yun Zhang, Jianlin Liu

**Affiliations:** 1 College of Pipeline and Civil Engineering, China University of Petroleum (East China), Qingdao, 266580 China; 2 College of Engineering, Lishui University, Lishui, 323000 China

**Keywords:** Biological physics, Mechanical engineering, Biomimetics, Engineering

## Abstract

With millions of years’ evolution, plants and fungi have developed a variety of ballistic dispersal structures for seeds or spores. One typical example is the catapult of an *Oxalis sp*., which can realize a consecutive seed ejection by triggering only one seed. If the protrusion on an aril, a specialized outgrowth covering a seed, is disturbed, cracks would occur and cause the opening of the aril. Subsequently, the whole aril snaps and transforms its stored strain energy to eject the inside seed with an optimal launching angle. Once the first seed is triggered, its curly aril will contact the next seed’s protrusion and induce its firing. This chain effect will further trigger the remaining seeds in turns, within 0.1 s. Inspired by this phenomenon, we invented a bionic ejection device to launch projectiles with high efficiency. This exploration is promising for a number of applications, such as drug delivery and oil displacement.

## Introduction

Although lacking muscles like animals, plants and fungi have evolved various functions to realize the organ deformation in the process of growth and reproduction^1–8^. Among them, mother plants or fungi can eject seeds or spores to certain places with the assistance of special structures, which are essential for the survival of species^9,10^. For example, when the falling liquid is grabbed inside the trumpet-shaped peridium of Nidulariaceae, the splashed droplets can carry the inner spores outside^11^. The coalescence of droplets near the Ballistospore can bend its supporting rod, i.e. sterigma, and release the strain energy to eject spores^12,13^. Some other plants or fungi could compress the water or air in their capsules for energy storage, to induce fluid jets for seeds or spores ejection, such as *Arceuthobium*, Coprophilous fungi *and Sphagnum fimbriatum*^14–17^. In addition, water evaporation can cause the geometrical incompatibility of the bilayers of some ballistic dispersal structures, such as the fern sporangium. The shrinkage of its outer annulus layer due to evaporation results in bending of the whole annulus. Then, the cavitation in the layer makes the annulus return its original geometry, finally emitting the elastic energy to eject spores^18,19^. The structure incompatibility is also beneficial for some plants to create quick coiling or buckling to eject seeds triggered by dehiscence^20–26^, such as *Erodium cicutarium*, *Tetraberlinia moreliana*, *Bauhinia* and *Equisetum* spores. Furthermore, some plants or fungi can eject seeds or spores with a lower energy cost. It can be observed that, due to the existence of partial cracks, the morphology of *Impatiens glandulifera* seedpod has been exquisitely designed to minimize the energy dissipation^27^. Another way for the far ejection of spore is to reduce the air dragging force; for example, *Sphagnum fimbriatum* is decorated with vortex rings^16^. Ascomycete fungi have evolved the optimal spore shapes, which own the minimum drag forces for prescribed volumes. Roper *et al*. numerically proved this fact by minimizing the ratio between the drag to mass^28^. Besides these, many plants, such as *Acanthaceae*^23^ and *Hura crepitans*^29^ can launch seeds with optimal angles to reach the distances up to several meters and tens of meters, respectively.

It should be mentioned that the adjacent catapults of the above-mentioned plants or fungi do not affect each other, i.e. the seeds or spores in one catapult are ejected just by their host catapult. This means that in order to launch the seeds or spores totally, all of the corresponding catapults need to be triggered. Different from these characteristics, we have investigated an *Oxalis sp*. that possesses a special ballistic dispersal structure to form a consecutive seed ejection, which consumes very little energy by triggering the first seed, similar to a domino show. Based on the morphology and location of collection, we think this species is *Oxalis corniculata*, however, we were not able to definitively ascertain the species. As a widely spread plant^30–32^, the *Oxalis sp*. can also launch seeds with an optimal angle, but the reason is distinct from the other plants or fungi. Therefore, the current study is directed towards a comprehensive understanding of the seed ejection mechanism of the *Oxalis sp*., especially on the energy transformation, optimal launching angle and consecutive ejection.

## Results

### A single seed ejection

**Figure 1:**
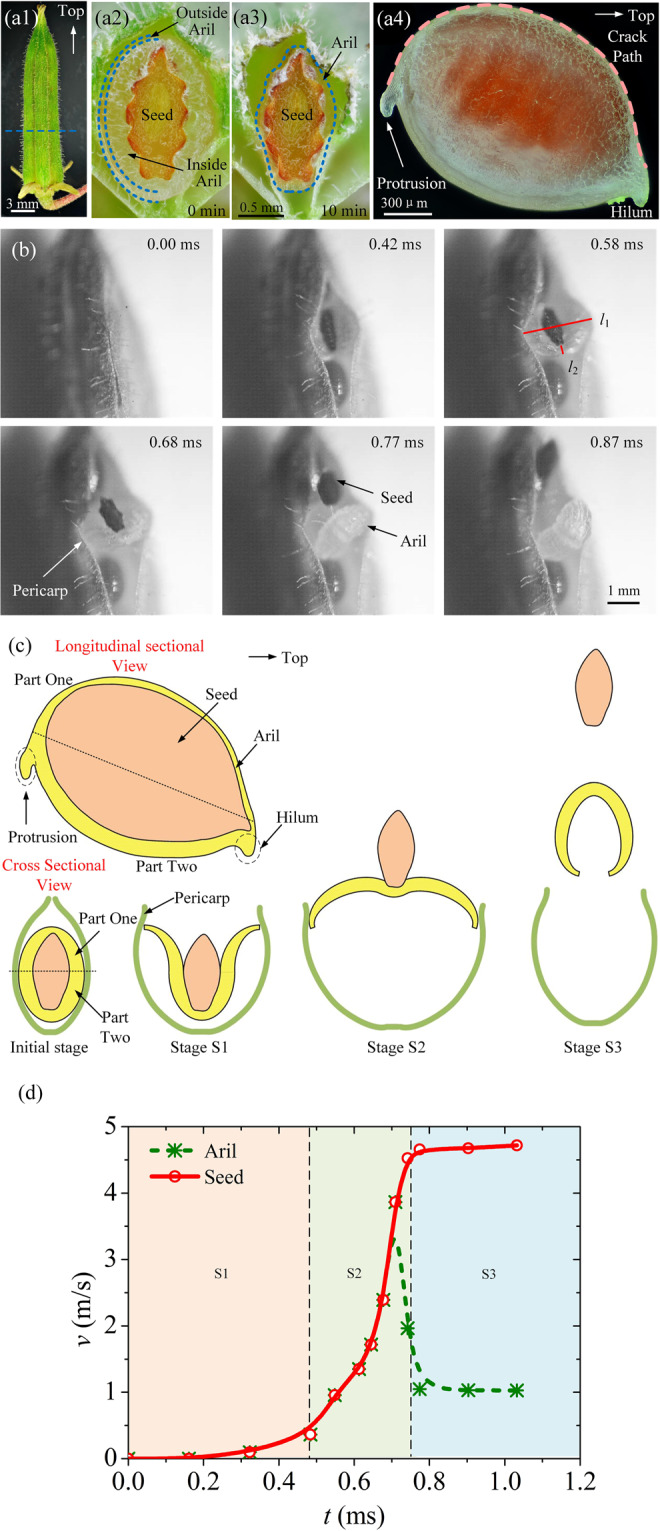
The process of a single ejection. (**a**) An *Oxalis sp*. fruit is shown in (a1). Its cross sectional view (a2) can be obtained by cutting the fruit along the blue dash line in (a1), where the blue dash lines in (a2) represent the boundaries of the outer and inner layers of aril. By adjusting the origin picture, the difference between the two layers of the aril can become clearer in Fig. S1 of Supplementary Materials. After 10 minutes, the aril will shrink as shown in the cross sectional view (a3). The 3D view of the whole seed with aril is displayed in (a4), with the orange dash line representing the crack path. The outer aril is smooth. (**b**) The photographic sequence of the seed ejection extracted from a high-speed video (see the Supplementary video S1). The inner surface of the aril is rough, as shown in Fig. S2 of Supplementary Materials. The straight red lines *l*_1_ and *l*_2_ stand for the horizontal and longitudinal lengths of the curly aril, respectively. (**c**) The scheme of the ejecting structures and the whole ejection stages. The longitudinal sectional view is the 2D view of (a4), and the cross sectional view is the 2D view of (a2). (**d**) Velocity versus time for the seed and aril in the ballistic dispersal process. The red solid line stands for the velocity of the seed, and the green dash line represents that of the aril.

We first consider the real seed structure in Fig. 1a. The fruit of the *Oxalis sp*. is composed of five valves equally distributed around a central axis, where each of them contains several seeds in the pericarp, and every seed is covered with an aril as shown in a4 of Fig. 1. We cut the seed along the blue dash line in a1 of Fig. 1, where the cross sectional view is a2 of Fig. 1.

The process of one single seed ejection of the *Oxalis sp*. is demonstrated in Fig. 1b (see the Supplementary video S1). It can be observed that there are a protrusion and a hilum (schematized in Fig. 1c) at both ends of the seed. There is a connection line (the straight dot line between the protrusion and the hilum in Fig. 1c) divides the seed into two halves, i.e. part one and part two. Based on this fact, it would be more convenient to define and describe the various stages of the single ejection process in the next part. Part one of the ellipsoidal seed is near the pericarp dehiscence (an already existing fracture).

As demonstrated in Fig. 1b, when the time *t* is in the range of 0 to 0.58 ms (stage S1 schematized in Fig. 1c), the crack initiates and propagates in part one of the aril, but only part one curls outwards and part two still attaches to the seed. During this period, the seed is propelled by the aril, with a slow speed increase. The curling of the aril is beneficial to open and expand the pericarp dehiscence, which makes room for the inside seed ejection.

From *t* = 0.58 to 0.77 ms (stage S2 schematized in Fig. 1c), part one of the aril supports the opened pericarp dehiscence and is fixed there, and then part two snaps rapidly within 0.2 ms as a thin shell. It is also noticed that the inner surface of the aril is rough and the outer is smooth. At this time, the aril surface in Fig. 1b is rough, which implies that the aril has snapped through with the curvature change from positive to negative. As a result, the velocities of the seed and the aril in this stage both increase abruptly. When *t* = 0.77 ms, they totally separate. After the snapping of the aril (stage S3 schematized in Fig. 1c), the seed and aril separate and both fly away from the fruit. The three stages including S1, S2, and S3 are displayed in Fig. 1d. It can be found that in S2, the velocity of the aril has already decreased before separation, implying that most of its stored strain energy is transformed into the kinetic energy of the seed, and the seed attains a peak velocity *v* = 4.7 m/s.

Let’s consider the origin of the driving force on the dispersal structure. As the aril is translucent (shown in a4 of Fig. 1), and the seed surface is dark red. Therefore, the aril exactly reflects the seed surface color. As the aril thickness increases, the color of the seed surface will be easier to observe, and the color becomes darker. This implies that the aril’s thickness increases from part one to part two. From a2 of Fig. 1, it is observed that the aril consists of two layers marked by blue dotted lines, where the outer layer is compact and thinner, and the inner layer is much thicker. In practice, Zhang *et al*. also stated that there are two layers in the young tissue of the aril from a cellular perspective^32^. When the cross section is exposed to air for 10 minutes, the whole aril will lose water to shrink and cling to the seed surface due to water evaporation, as shown in a3 of Fig. 1. It can be inferred that there is a lot of water in the inner layer according to Edwards *et al*.^33^. They declared that the water absorption of the inner layer can cause it to expand, while the outer layer is resistant to water uptake due to its high lipid content and will remain unchanged. This geometrical incompatibility between two layers will produce a prestrain in the outer layer ${\varepsilon }_{p}$, inducing compressive stress in the inner layer and a tensile stress in the outer one. In addition, the difference in swelling between the two layers can be inferred from other structural information. It is well known that, if the two layers swell at the same rate, there will be no prestrain in the mature aril. During this situation, the aril will stay still when the seed is cut from the middle. Actually, after being cut, the aril tends to quickly snap and curl towards the outer layer, which indicates that the mature outer layer owns tensile strain and the inner layer owns compressive strain. Thus, these two layers swell differently as the seed matures, which will generate the strain energy in the mature aril. If this kind of stored strain energy is released, it will become the driving force to push the seeds outside.

Next, we make a mechanistic analysis of how much kinetic energy the seed can obtain, with respect to the curling of the aril. A characteristic length is introduced, i.e. *L* = $\sqrt{{l}_{1}{l}_{2}}$, where *l*_1_ and *l*_2_ (measured by Image J) stand for the horizontal and longitudinal lengths of the curly aril shown in Fig. 1b. The quantity *L*^2^ is used to describe the area of the aril that have curled, which stands for the bulking extent of the aril. The elastic energy *E*_e_ $\sim E{L}^{4}\kappa$, where *E* is the aril modulus, $\kappa$ is the aril’s average curvature; and the kinetic energy *E*_k_ $\sim m{v}^{2}$, where *m* is the seed mass. Here, we use the dimensional analysis to obtain the target formula through the good choice of the dimensioned physical variables that are relevant to the problem^34^. When the aril’s average curvature varies from the original one ${\kappa }_{0}$ to the current one $\kappa$, the velocity of the seed accelerated by the released elastic energy is scaled as

1 $$v={L}^{2}\sqrt{\frac{{E}_{o}\kappa }{{m}_{1}}}f\left(\frac{{m}_{2}}{{m}_{1}},\frac{{E}_{i}}{{E}_{o}},\frac{{\kappa }_{0}}{\kappa },\kappa {h}_{o},\kappa {h}_{i},{\varepsilon }_{p}\right),$$

where *h*_*o*_ and *h*_*i*_ are the thickness of the outer and inner layers, respectively; *E*_*o*_ and *E*_*i*_ are Young’s moduli of the outer and inner layers, respectively; *m*_1_ and *m*_2_ are the mass of the seed and aril, respectively.

**Figure 2:**
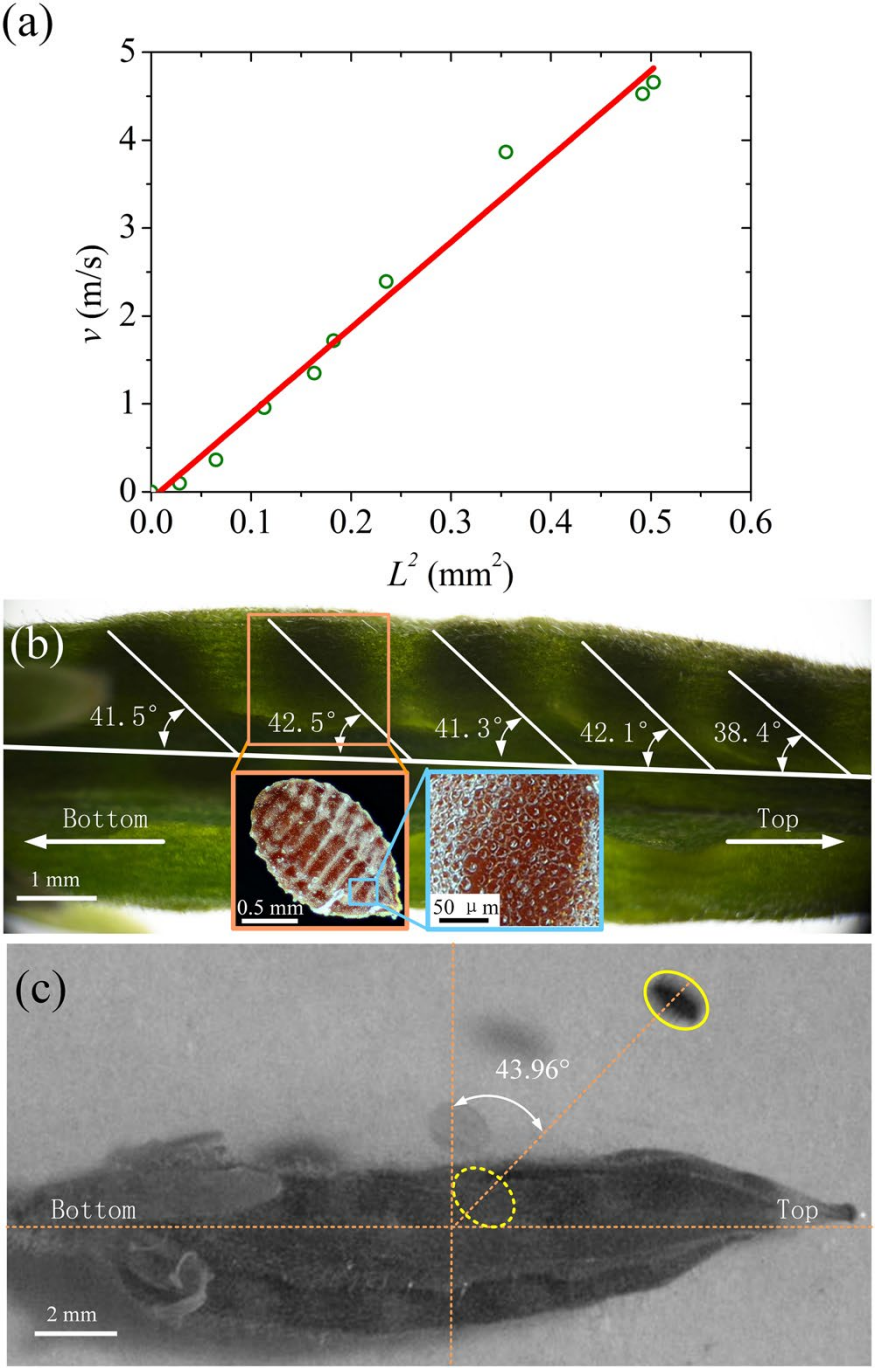
The mechanism of ejection energy translation and the forming of the optimum angle. (**a**) Linear curve of the seed velocity versus the parameter *L*^2^. In the picture, the blue circles represent real experimental data, and the red straight line is a fitted linear curve. (**b**) Seed location in the fruit and the multilevel structure of seed surface. The seed location is described by the angle between the connection line of the seed and the axis of fruit, marked with white lines. The seed surface has several parallel grooves and smaller round convex structures. (**c**) Real launching angle of the seed. The upright line vertical to the long fruit represents the real horizontal direction.

It is noticed from Fig. 1b that, the curvature of the curled aril changes slightly, so the value of $\kappa$ can be viewed as a constant. Figure 2a shows that the velocity of the seed is proportional to *L*^2^ when the other parameters are prescribed. The whole value of *L* can be obtained by measuring the horizontal and longitudinal lengths of the curly aril in every picture during the ejection process. To determine the coefficient *f*, the data fitting is made, and the value of *R*^2^ on the curve is 0.96. The slope of the fitted line is 9.74 $\times$ 10^6^ m^−1^s^−1^, where the measured parameters are given as follows: *m*_1_ = 0.287 mg, $\kappa$ = 6127.67 m^−1^, *E*_*o*_ is 100 MPa^26^, and thus the factor can be calculated as *f* = 0.0067.

Although the seed of the *Oxalis sp*. can get enough kinetic energy from the catapult, its launching angle should be optimized to reach the farthermost destination. It is observed in Fig. 2b that the angle between the connection line (from the hilum to the protrusion) of the seed and the axis of fruit (usually vertical to the horizontal surface) has the average value $\beta$ = 41.2 $\pm$ 1.6 ° by measuring these 5 angles. Since uncovering the pericarp will affect the original seed position, it may be a better choice to use the entire fruit for measurement based on the seed shadow. In addition, it is noticed that there are several parallel grooves on the seed surface (vertical to the seed connection line, shown by the orange frame), whose width and depth are 157.4 $\pm$ 13.6 ${\rm{\mu }}{\rm{m}}$ and 70.1 $\pm$ 14.1 ${\rm{\mu }}{\rm{m}}$ by measuring 7 grooves, respectively. The surface of the seed is very rough distributed with numerous round convex structures with a diameter of 9.8 $\pm$ 1.0 ${\rm{\mu }}{\rm{m}}$ by measuring 8 structures, which is shown by the blue frame. Meanwhile, there are grooves on the inner surface obviously in Fig. S2,b, which are sculptured negatively by the grooves on the seed surface. Figure 1a2 also shows that these grooves on the aril inner surface can match well with the seed rough surface. This multilevel structure will enhance the friction on the seed surface and prevent it from slipping during ejection. This locking mechanism in the friction theory may improve the conversion efficiency from the strain energy of the aril to the kinetic energy of the seed.

Moreover, it ensures that the velocity of the seed is prone to the parallel grooves, in which case $\beta$ is exactly the intended launching angle according to their geometric relationship. The function of this multilevel structure can be validated by Fig. 2c, where the actual launching angle of the seed is 45.0 $\pm$ 7.0° measured by Image J based on 5 seeds (it is 43.96° in the figure), which is very close to the value of $\beta$. In theory, if we consider the air resistance^29,35^, the height of the fruit ranging from 2 to 18 cm and the ejection velocity of the seed *v* = 4.7 m/s in Fig. 1d, the optimal projection angle $\alpha$ to reach a longest ballistic dispersal distance would be in the range of 42.0–44.0° (See Supplementary Materials), which is consistent with our observation.

### Consecutive ejection as a domino show

For a fruit with many seeds, the *Oxalis sp*. can eject their seeds by consuming very little energy, which is more complex than one single seed ejection. the *Oxalis sp*. achieves this goal just by triggering the first seed, leading to a consecutive ejection like a domino show. The first concern on the consecutive ejection is how the seeds are triggered, and how the crack on the aril initiates.

**Figure 3:**
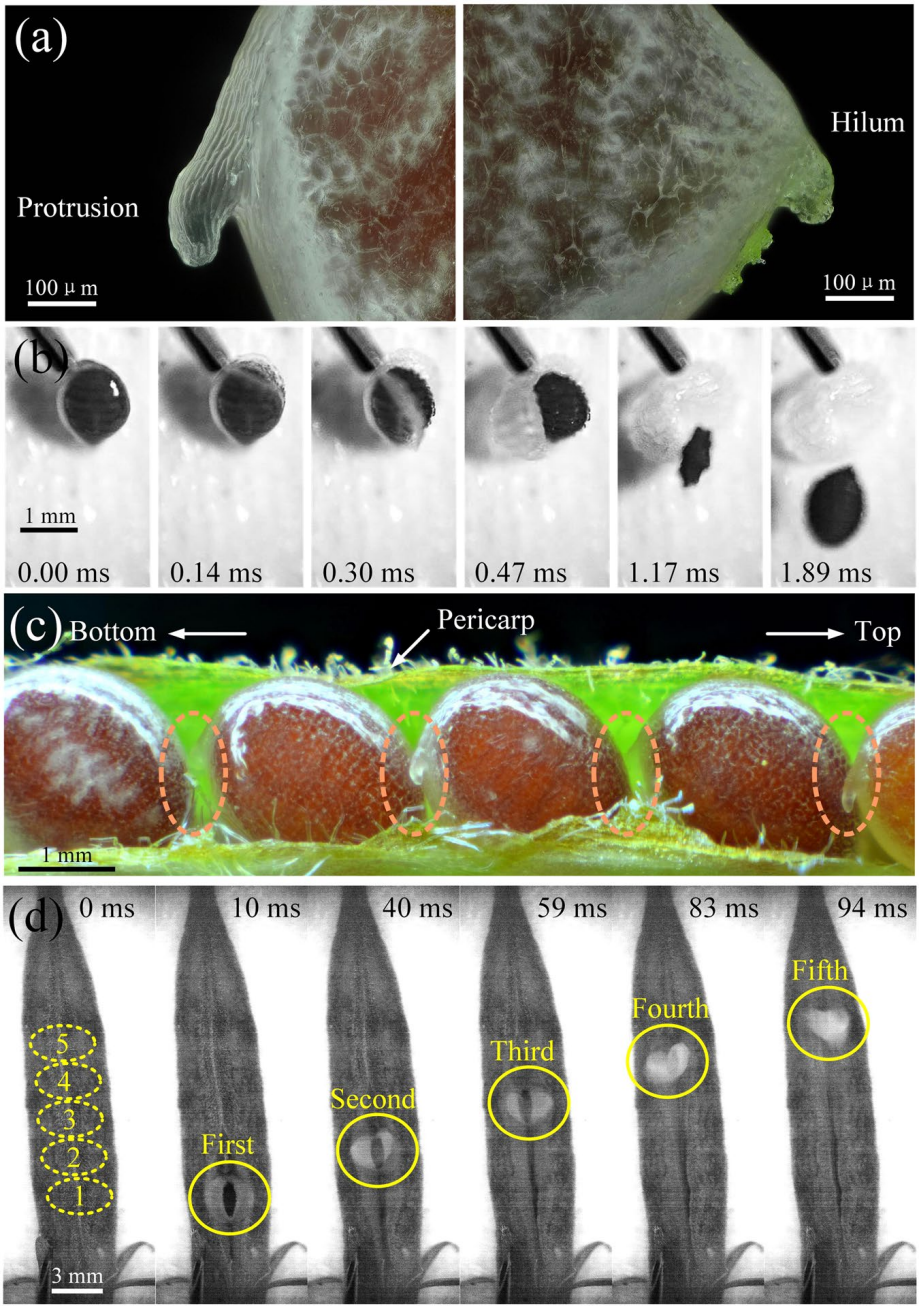
Consecutive ejection of seeds in fruit. (**a**) The protrusion and hilum on the seed. In the right picture, the protrusion looks like a hook tube with many grooves on its surface. Due to the translucent nature of the aril, the white color of the hilum indicates that the hilum is thick. (**b**) Photographic sequence of the crack initiation and propagation triggered by a syringe needle (see the Supplementary video S2). Only when the protrusion is touched, can the crack occur and propagate along the orange dash line in a4 of Fig. 1. (**c**) Seed arrangement in one valve. The position of the protrusion is located near the front seed, which plays a key role in the continuous ejection. (**d**) Photographic sequence of the consecutive ejection of five seeds in one valve (see Supplementary video S3). The five seeds are ejected from the bottom to the top of the fruit within 0.1 s.

As shown in Fig. 3a, the hilum of the aril is thick with low stress. In this case, the strain energy stored here may not be sufficient to support the propagation of the crack, and the crack will be hindered at the hilum. The protrusion looks like a hook tube, whose surface is distributed with many grooves. As shown in Fig. 3b (see Supplementary video S2), when a syringe needle is used to touch the areas except for the protrusion, the seed fails to eject. Only when the tip of the protrusion is touched by the needle, can the crack initiate and develop along the edge of part one. When the tip of the protrusion is loaded, the protrusion can be regarded as a cantilever, whose fixed end will produce the maximum stress. Due to the stress concentration, the grooves at the end of protrusion are most prone to collapse, i.e., the crack will occur here, which also is proved by simulation^37,26,36^^,^ (see Supplementary Materials). From a2 and a4 of and Fig. 1, we can see that the aril is the thinnest along the crack path, i.e., the orange dash line in a4 of Fig. 1. Due to the sharp edge of the seed, the inner surface of the aril has a sharp groove along the orange dash line. Therefore, when the aril is prestressed, the maximum stress is created due to this sharp groove, then the crack will propagate along the orange dash line in a4 of Fig. 1, inducing the snapping of the aril to eject the inner seed. Thus, we are able to speculate that the protrusion can act as the trigger for seed ejections, which will cause a series of seed ejections.

The arrangement of the red seeds inside the green pericarps of one valve can be observed in Fig. 3c, where the protrusion of each seed is close to its former seed. It is easy to speculate that when the first seed aril cracks and curls, it will contact the protrusion of the second seed, and induce a new triggering. This triggering can be transferred to the onset of the third, fourth, fifth, etc., and make all the seeds fully ejected. Normally, the first triggered seed is near the bottom of the fruit (shown in Fig. 3c), as the fruit can be viewed as a cantilever where the clamped end has the biggest bending moment and stress. The disturbances, such as wind, rain, and vibration, will be transported from the bottom to the top of the fruit (see the Supplementary video S3), and the adjacent seeds in the valve are consecutively ejected within 0.1 s (Fig. 3d). According to our statistics for 23 samples, the average time between two ejections is 27.11 $\pm$ 25.48 ms. The first triggered seed is usually the second or third seed from the bottom, which can be ejected later by the pericarp disturbance induced by its following seed ejections. The interconnected arils of all the seeds through protrusions make up an ejection device, which needs very little energy for solely triggering the first seed, -this is a typical example of the principle of least action. In practice, the ejections of fruit are not always the consecutive ejection, whose success rate is 74.36%. Even one of the seeds fails to eject by accident, the next seed can still restart to eject, as each one has a perfect catapult and can eject independently, as shown in Fig. 1b. Of course, the disturbance of the pericarp can also trigger the seed ejection of other valves, making the seeds in the fruit spread widely.

### Bionic ejection device

**Figure 4:**
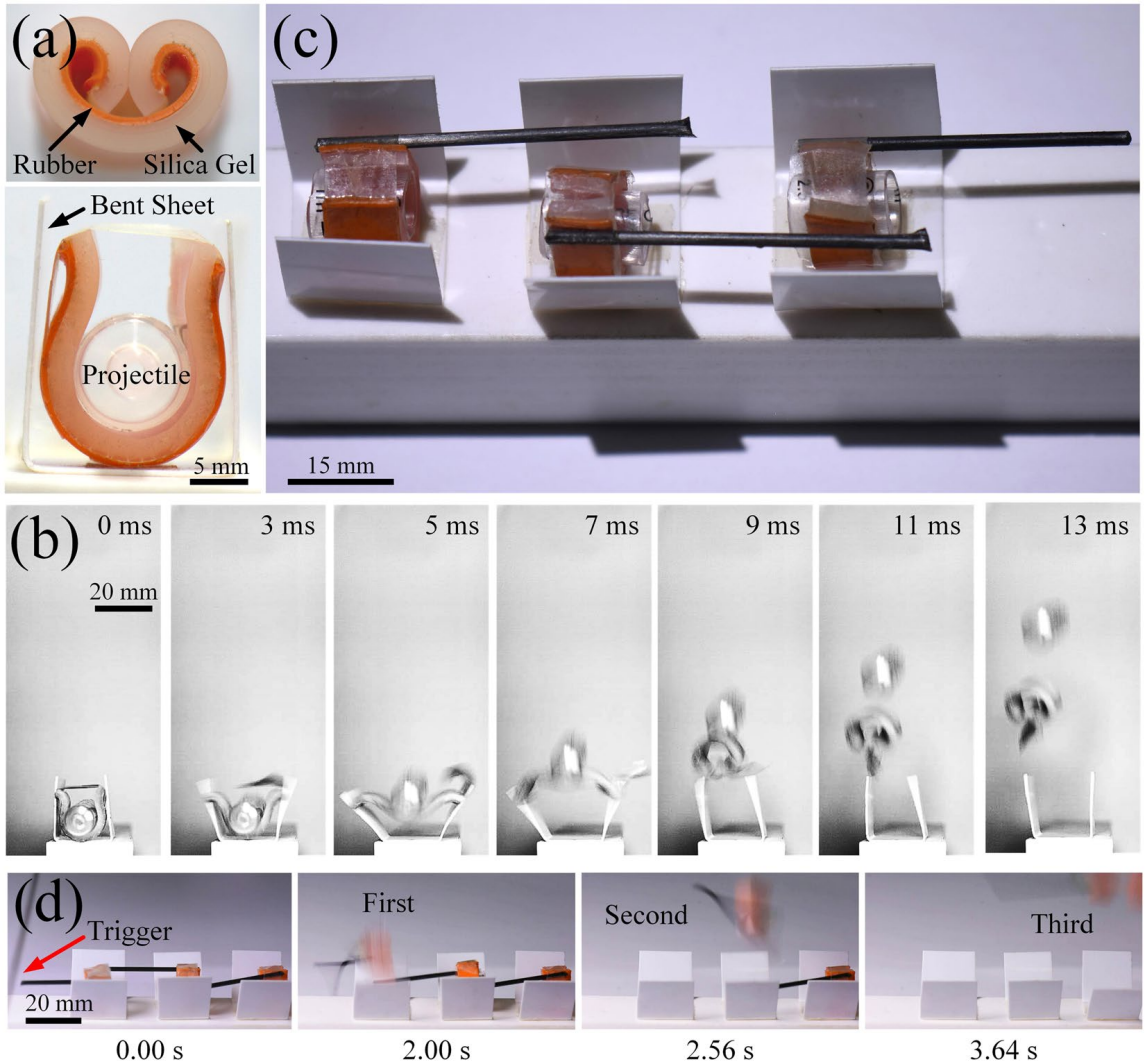
The bionic ejection device. (**a**) Bionic aril composed of a red rubber layer and white silica gel layer in the natural configuration in the top figure, and the bionic ejection device before ejection in the bottom figure. (**b**) Photographic sequence of the bionic ejection process. The two ends of the bionic aril will separate, open the white sheet, and curl outwards to eject the bionic seed, whose process is like the real seed ejection. (**c**) Consecutive bionic ejection device. The black rod is similar to the seed protrusion, which can help the bionic device to achieve the continuous ejection. (**d**) Consecutive ejection process (see the Supplementary video S4). When the first black rod is touched, the first bionic ejection device will eject the bionic seed, inducing the consecutive ejection process.

Inspired by the ejection mechanism of the *Oxalis sp*., a bionic ejection device can be invented. The bionic aril is made of a bilayer including a white silica gel and a red rubber, whose natural state is shown in Fig. 4a. The bilayer will bend towards the rubber layer in an equilibrium state due to the prestretched rubber layer. For one bionic catapult, a projectile made of a segment of needle tube is wrapped by the bilayer. The bilayer is forced to curl with the red rubber layer becoming the outer layer, and the two ends of the bilayer are fixed by an adhesive tape. Both of the bilayer and projectile are placed in the bionic pericarp, i.e. the bent polyethylene sheet fixed on the substrate.

When the tape peels under the prestress (similar to the crack initiation of a real aril), the two ends of the bilayer will separate and curl outwards, then open the polyethylene shell, as shown in Fig. 4b. This process is marked with S1, analogous to the first stage of the real seed ejection. Then in S2, the snapping of the bilayer rapidly ejects the projectile with the two ends of the bilayer supporting the bent sheet. In S3, the bilayer finally goes back to its natural configuration and flies away with the projectile from the bent sheet. The elastic energy of the bilayer is calculated as 32.77 $\pm$ 0.54 mJ^38–40^ based on 3 measurements, and this value matches the kinetic energy of the bilayer and the projectile very well, which reads 31.31 $\pm$ 0.26 mJ based on 11 measurements (See Supplementary Materials).

A rod between the adhesive tape and the bilayer is used to simulate the seed protrusion of the *Oxalis sp*.. When the rod is touched, it will peel off part of the adhesive tape, reducing the adhesion area. The adhesion force will drop and cannot resisit the force produced by the bilayer pre-strain. Eventually, the black rod will cause the tape to completely peel off and induce a bionic ejection as a trigger for the devices. Three bionic ejection units with rods form a consecutive catapult as shown in Fig. 4c. When the rod of the first bionic ejection unit is touched by the disturbance, the tape peels from the bilayer and triggers the ejection of the first unit, as shown in Fig. 4d. The curled bilayer will touch the rod of the second unit, leading to a consecutive ejection spreading from the left to right within 3.64 s (see the Supplementary video S4). Each unit is mainly triggered by the former adjacent one, and can also maintain the possibility to eject alone when the former fails to catapult.

## Discussion

The seed ejection mechanism of the *Oxalis sp*. has been explored well in this study. We first focus on a single seed ejection. When the seed starts to eject, the whole aril deforms suddenly and transforms its stored strain energy to eject the inside seed. The seed is obliquely arranged with respect to the fruit axis, and its surface has multilevel structures, which can make it launched with an optimal angle to reach the farthermost destination. In this ejecting process, the time’s scale is 0.77 ms, and the smallest dimension of the aril is 1.21 mm in a4 of Fig. 1. In that case, the *Oxalis sp*. just belongs to the explosive fracture dominated zone illustrated by Skotheim^3^. Meanwhile, it is close to the snap-buckling dominated zone, which may be due to that the aril will also snap in the seed ejection.

We later explore the consecutive ejection of the *Oxalis sp*. as a domino show. The special structure on the aril, i. e. protrusion, is the key role in this process. When the protrusion is disturbed, it would cause the opening of the aril. Once the first seed is triggered, its curly aril will contact the next seed’s protrusion and induce its firing. This chain effect can trigger the onset of the remaining seeds in proper order. Thus a bionic ejection device is invented to launch projectiles with high efficiency. This investigation has important implications in drug delivery, mechanical engineering and oil displacement, etc. For instance, it is a challenge to deliver surfactant to the underground zone full of residual oil due to the adsorption of rock surfaces. A structure similar to the seed can be used to achieve the goal by ejecting the inner surfactant triggered by the disturbance of oil-water surface tension when the structure encounters with the residual oil.

## Materials and Methods

### Ejection experiments

The *Oxalis sp*. in the experiment grows naturally in the Tangdao bay park located in Qingdao of China. Some ripe fruits are selected for the ejection experiment, and they are vertically fixed on the test-bed in our lab. A syringe needle is used to touch the bottom of the fruit, and the ballistic seed dispersal process is recorded with a high-speed camera (Phantom V2512 bought from Vision Research Company). Figures 1b, 2c, 3b,d and 4b are recorded by Phantom V2512 with 31000, 25000, 70000, 1000 and 1000 f/s, respectively. Figure 4d is recorded by Nikon D720 with 25 f/s.

In the bionic ejection experiment, a red rubber with a thickness of 0.76 mm, silica gel pad with a thickness of 2 mm and polyethylene sheet are bought from the Dao Guan Ship in Tmall. The rubber layer with width 9.0 mm and length 22.8 mm is stretched to 44.0 mm and stuck to the silica gel layer with width 9.0 mm and length 44.0 mm by superglue HJ-403 from Dongguan Gu Bang Adhesive Co., Ltd. The rubber layer prestrain 44/22.8–1 = 0.93. The bilayer and the bionic ejection devices are photographed by a camera (Nikon D720). All the experiments are conducted under a temperature of 25 °C.

The pictures of the aril and seed are captured by the extended depth-of-field microscope, LY-WN-YH3D from Chengdu Li Yang Precision Machinery Co. Ltd. The picture analysis is through a freeware ImageJ. The mass of the seed, bilayer and needle tube are measured by an electronic balance (PT-405 from Polestar Scientific Instrument China, with 0.01 mg accuracy). The Young’s moduli of the white silica gel and a red rubber are measured in a quasi-static state with a strain rate of 0.0019 s^−1^ by the universal testing machine UTM–1432 from Chende JinJian Testing Instrument Co., Ltd.

## Supplementary information

Supplementary Video S1

Supplementary Video S2

Supplementary Video S3

Supplementary Video S4

Supplementary Materials

Supplementary Video Information
