# Supplementary material for: Seed ejection mechanism in an Oxalis species
Source: Sci Rep. 2020 Jun 1;10:8855. doi: 10.1038/s41598-020-65885-2 (PMC7264325; doi:10.1038/s41598-020-65885-2)
Supplement: Supplementary file 5 — Supplementary Materials [file 41598_2020_65885_MOESM5_ESM.pdf]

Supplementary Materials

Seed ejection mechanism in an *Oxalis* species

Shanpeng Li[1, 2], Yun Zhang[1], Jianlin Liu[1*]

1 *College of Pipeline and Civil Engineering, China University of Petroleum (East China), Qingdao 266580, China*

2 *College of Engineering, Lishui University, Lishui 323000, China*

Two layers in aril

To make a2 in Figure 1 clearer, we can further adjust the contrast, saturation, and hue of the image. As shown in Figure S1, the difference between the two layers is clear, which proves that there are indeed two layers in the aril. The outer surface of the aril is smooth, while the inner surface is rough, as shown in Figure S2.

Optimal launching angle calculation

Considering the air resistance, the horizontal dispersal distance S of the seed[35] is expressed as

$$S = \frac{1}{k}\ln\left(1 + tkv\cos\alpha\right), \tag{1}$$

where $k = C_D \rho A / \left(2m_1\right)$, the air resistance coefficient[29] C_D=0.087 m^{-1}, the air density is ρ=1.3 kg m^{-3}, the cross sectional area of the seed is A=0.52 mm^2, and the flight time of the seed t is given as

$$t = \frac{1}{\sqrt{gk}}\left[\tan^{-1}\left(\frac{v\sin\alpha}{v_T}\right) + \ln\left(e^{kH_1} + \sqrt{e^{kH_1} - 1}\right)\right], \tag{2}$$

where the maximum height H_1 reached by the seed is

* Corresponding author: Jianlin Liu. E-mail: liujianlin@upc.edu.cn.

$$H_1 = H_2 + \frac{1}{2k}\ln\left(1+\frac{v^2\sin^2\alpha}{v_T^2}\right). \quad (3)$$

In A3, the parameter H_2 is the height of the fruit ranging from 2 to 18 cm, the terminal vertical velocity of the seed in free falling is $v_T = \sqrt{g/k}$, and the gravitational acceleration is g=9.8 m/s^2. As a consequence, the function curves between the ejection distance and the launching angle can be given, as shown in Figure S3.

Crack simulation. The protrusion is very small, and once touched, it will burst and curl within one millisecond. It is very difficult to explore the actual structure inside of the protrusion. Based on this fact, the FEM can be used as a numerical experiment to demonstrate this process, even though the model may not be exactly the same as the actual structure of the protrusion.

The numerical simulation in Figure S4 indicates that cracks occur in the grooves near the base of the protrusion, and the existence of cracks will create the condition for the propagation of the cracks. We use the crack simulation to prove that the protrusion is just the trigger for the seed ejection, where the protrusion is a hook-tube structure of aril. It is well known that when the stored elastic energy is greater than the fracture energy, existing cracks will propagate. If there exists no crack on the protrusion, the crack propagation will not happen even if the stored elastic energy is greater than the fracture energy. In that case, the aril cannot snap to eject the inner seed. To obtain a more convincing result, here, we use the extended finite element method (XFEM) in ABAQUS 6.14 to study the initiation of a crack during the deformation of protrusion. The diameter and thickness of the protrusion are measured as 91 ± 2.9 μm and 6.4 ± 0.3 μm, respectively, and there are many grooves on its surface with the width being 10.4 ± 0.9 μm and depth being 4.4 ± 0.8 μm based on 4 measurements. The aril with about 1.5 mm is very small, and the aril will become dry very quickly in several minutes. Thus, the aril modulus is hard for our current devices to measure, and we use the modulus of 100MPa[26] to simulate the crack initiation. Because, four elaters in this

reference, the flexible ribbon-like appendages of the spore, own bilayer and work to eject the spore, whose function is similar to that of aril. Meanwhile, the scale of the elater is about 0.2 mm, which is close to that of the aril. Thus, the parameters are chosen as follow: Young's modulus is 100 MPa[26], Poisson's ratio is 0.25[36], maximum principal stress is 0.76 MPa, and fracture energy[37] is 152 J/m^2. The base of the protrusion tube is modeled as a clamped end. The tip of the protrusion is loaded with a displacement of 0.007 mm, which is vertical to the clamped end. The tube is meshed with C3D8R elements, whose number is 70720.

A single bionic ejection unit calculation

To qualify the process in Figure 4b, the process can be analyzed from the view of energy. The velocity curves are plotted in Figure S5, implying that the kinetic energy of the system mainly comes from the stage S2. The whole kinetic energy of the system reads

$$E = \frac{1}{2} m_3 v_3^2 + \frac{1}{2} m_4 v_4^2, \tag{4}$$

where the mass of the bilayer and projectile are m_3=1.17 g and m_4=0.62 g, and the velocity of the bilayer and projectile are v_3=7.76 m/s and v_4=4.64 m/s, respectively. Thus the kinetic energy can be calculated as 31.31 mJ, which comes from the elastic energy of the bilayer based on the principle of energy conservation.

To calculate the strain energy stored in the bilayer, we first build a Cartesian coordinate system {O, x, y} schematized in Figure S6, where x axis is on the interface between the rubber and silica gel layer and along their longitudinal direction. This bilayer is considered to satisfy the Euler-Bernoulli postulation, and the neutral axis is not on the x axils due to the bilayer structure[38]. The stress at an arbitrary point (x, y) of the rubber layer is

$$\sigma_r = E_r\left(\varepsilon_p - \varepsilon_0 - \kappa y\right), \tag{5}$$

where Young's modulus of the rubber is measured as E_r=1.43 MPa, ε_p=0.93 is the

prestrain of rubber layer, $\varepsilon_0 = \kappa\Delta$ is the axial strain, κ is the curvature of the neutral axis, and Δ is the distance of the neutral axis to *x* axis. The stress of the silica gel layer is

$$\sigma_s = -E_s\left(\varepsilon_0 + \kappa y\right), \tag{6}$$

where its Young's modulus is measured as E_s=3.93 MPa. The equilibrium equations about the force and moment on the cross section read

$$\int_{A_r} \sigma_r \mathrm{d}A_r + \int_{A_s} \sigma_s \mathrm{d}A_s = 0, \tag{7}$$

$$\int_{A_r} y\sigma_r \mathrm{d}A_r + \int_{A_s} y\sigma_s \mathrm{d}A_s = 0, \tag{8}$$

where the cross sectional area of the rubber and silica gel layer are $A_r=bh_r$ and $A_s=bh_s$, the thickness of them are h_r=2 mm and h_s=0.76 mm, respectively. By deleting the quantity ε_0, one has

$$\kappa = \frac{6E_r E_s \varepsilon_p h_r h_s\left(h_r + h_s\right)}{E_r^2 h_r^4 + E_s^2 h_s^4 + 2E_r E_s h_r h_s\left(2h_r^2 + 2h_s^2 + 3h_r h_s\right)}. \tag{9}$$

When the rubber thickness is close to zero, i.e. $h_r / h_s =$ 1, the curvature of the neutral axis can be reduced to

$$\kappa \approx \frac{6\sigma h_r}{E_s h_s^2}, \tag{10}$$

where $\sigma = E_r \varepsilon_p$ is the mismatch stress of the two layers. Eq. S10 is just the famous Stoney formulas[39,40], which in turn verifies the correctness of the above derivation. By deleting the quantity ε_p, one has

$$\Delta = \frac{E_r h_r^3 + E_s h_s^2\left(3h_r + 4h_s\right)}{6E_s h_s\left(h_r + h_s\right)}, \tag{11}$$

which is calculated to be 1.25 mm. Finally, the elastic energy of bilayer can be written as

$$E = \int Mb\mathrm{d}\theta,$$

$$= \frac{b}{12s}\left[3\Delta\left(E_s h_s^2 - E_r h_r^2\right) - 2\left(E_s h_s^3 + E_r h_r^3\right)\right]\theta^2 + \frac{bE_r\varepsilon_p h_r^2}{2}\theta \tag{12}$$

where s is the arc length of the bilayer, θ is the rotation angle of the bilayer. We divide the whole bilayer into three segments. The main part of the bilayer is the lower part, which is close to the projectile before ejection. The length of this segment is measured as 18.53 mm, whose rotation angle changes from $-\pi$ to 0.85π after ejection. The remaining two parts are just the upper parts of the curved bilayer, whose lengths are both equal to 12.74 mm. Their rotation angle changes from 0 to 1.33π. As a result, the elastic energy of the bilayer is calculated as 32.87 mJ. Considering the energy dissipation, the calculated result fits very well with the kinetic energy of 31.31 mJ, which validates our model based on the ejection mechanism of the *Oxalis sp.*

Figures

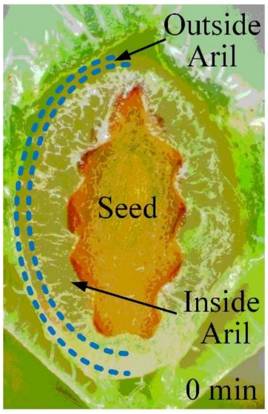

(S1) The cross sectional view obtained by cutting the fruit along the blue dash line in a1of Figure 1. The aril zone between the two blue dash lines is the outer layer, and the other zone of the aril is the inner layer.

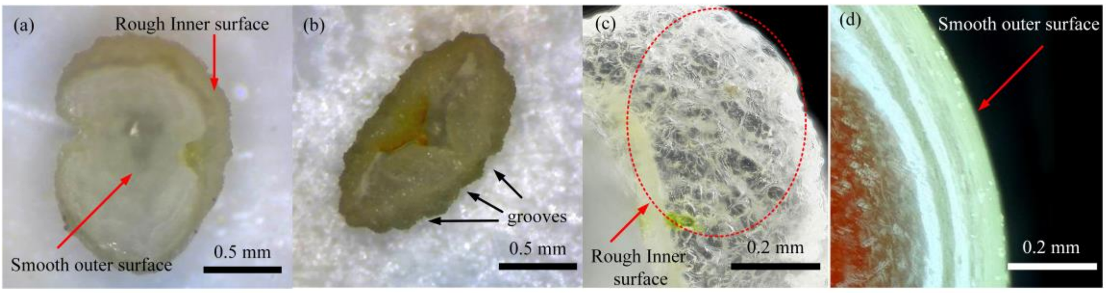

(S2) The aril surface morphology. The outer surface of the aril is smooth, while the inner surface is rough. (a) implies that the aril has snapped during ejection. (b) shows an aril after snapping with a higher buckling degree. There are grooves on the inner surface, which are sculptured negatively by the grooves on the seed surface. (c) shows the rough inner surface of the aril after snapping at a high magnification. (d) shows the smooth outer surface of the aril before snapping at a high magnification.

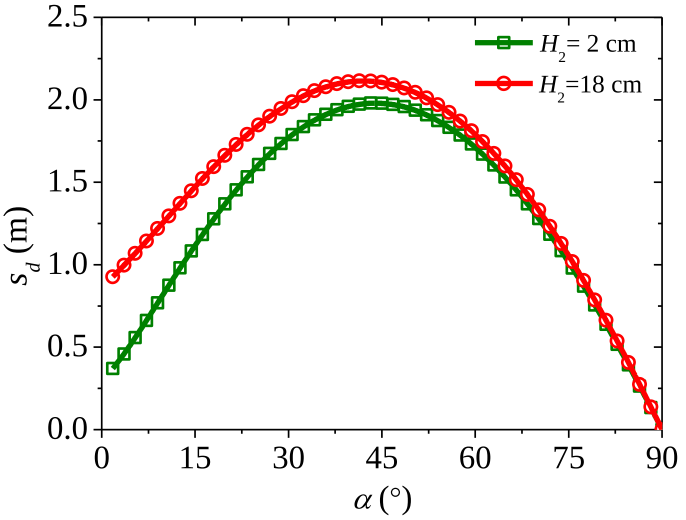

(S3) the function curves between the ejection distance and the launching angle. The red line stands for the height of 18 cm, and the green line represents the height of 2 cm.

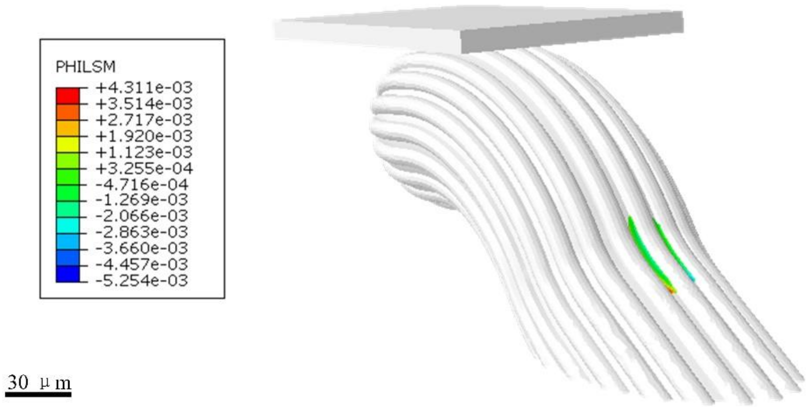

(S4) Numerical simulation of the crack initiation on the protrusion under a vertical displacement. The PHILSM in the legend is the signed distance function to describe the crack surface. The crack surface is situated in the elements where the value of PHILSM transitions from a negative number to a positive number. The zones with green color stand for the existing cracks.

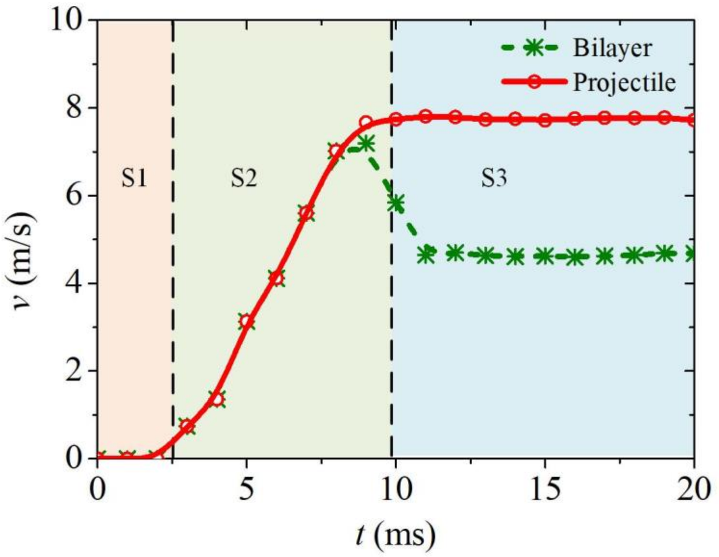

(S5) The velocity curves of the bionic device. The red solid line stands for the velocity of the bionic seed, and the green dash line represents that of the bionic aril.

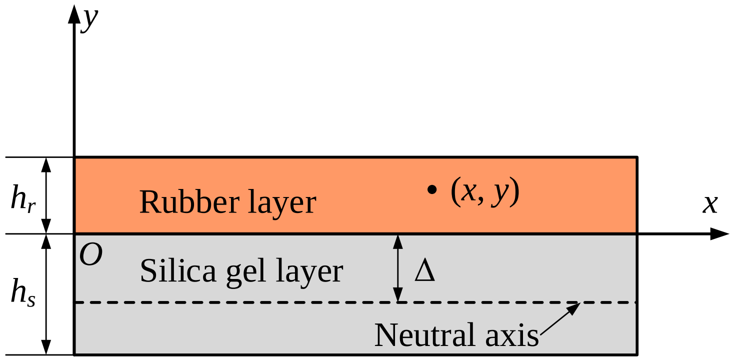

(S6) The Cartesian coordinate system of bilayer. The orange area stands for the rubber layer, and the gray zone represents the silica gel layer.
