# Supplementary material for: Seed ejection mechanism in an Oxalis species
Source: Sci Rep. 2020 Jun 1;10:8855. doi: 10.1038/s41598-020-65885-2 (PMC7264325; doi:10.1038/s41598-020-65885-2)
Supplement: Supplementary file 6 — Supplementary Video Information [file 41598_2020_65885_MOESM6_ESM.docx]

**Supplementary data.**

Supplementary materials calculating the optimal ejection angle of seed and analyzing the process of the bionic ejection.

Supplementary video S1 presenting the ejection process of a single seed in the pericarp of the *Oxalis sp.* Supplementary video S2 presenting the crack initiation and propagation of a single seed*.* Supplementary video S3 presenting a consecutive ejection of five seeds in one valve*.* Supplementary video S4 presenting a consecutive ejection of three bionic seeds*.*
